# Adaptive Suppression of MAPT Transcription Maintains Tau Proteostasis in Developing Human Neurons

**DOI:** 10.21203/rs.3.rs-7585327/v1

**Published:** 2025-10-03

**Authors:** Mallory R. Shin, Narges Firouzshahi, Gage Liddiard, Megan Weis, Jacob Simmering, Joseph Glykys, Mark Schultz, Marco M. Hefti

**Affiliations:** University of Iowa; University of Iowa; University of Iowa; University of Iowa; University of Iowa; University of Iowa; University of Iowa; University of Iowa

**Keywords:** proteostasis, Alzheimer’s disease, tau protein (tau), autophagy, neuroprotective, ubiquitin-proteasome system, neuron, neurodegeneration, induced pluripotent stem cell (iPS cell) (iPSC), tauopathy, neurodevelopment

## Abstract

Developing human neurons express abundant tau yet show little toxicity, suggesting built-in mechanisms that restrain tau when protein clearance falters. We combined human tissue analyses with cell-based perturbation assays to define this response. In iPSC-derived forebrain neurons, brief proteasome blockade with epoxomicin (0.25 μM, 24 h; n=3/condition) triggered a coordinated transcriptomic program: hierarchical clustering of RNA-seq data resolved two opposing modules—an up-regulated proteostasis module (ubiquitin-proteasome, autophagy-lysosome, chaperone-mediated folding) and a down-regulated MAPT-linked neuronal/energetic module (microtubule/organization/transport, synaptic signaling, oxidative phosphorylation). MAPT transcripts decreased by both PCR and RNA sequencing in proteasome but not-autophagy impaired neurons even though tau protein levels decreased in both. Expressing tau from a constitutive promoter bypassed this transcriptional brake and increased tau during proteasome inhibition. Qualitative confocal imaging of human cortex (fetal, adult control, and Alzheimer’s disease) showed tau locally nested within proteasome-positive regions with partial overlap with lysosomes, consistent with increased quality-control engagement when tau burden is high. To nominate regulators coupling proteostatic stress to MAPT repression, we integrated promoter motif enrichment (HOMER), transcription-factor enrichment from curated libraries (ChEA3; MeanRank on up/downregulated sets), and JASPAR scanning of the MAPT promoter. A consensus highlighted E2F1, EVT1, Lhx1, and TCF3 among top candidates for MAPT regulation. Together, these data support a proteostasis-first adaption in which neurons activate quality-control programs while transcriptionally reducing MAPT and allied neuronal demands, offering transcription-factor targets and a framework for modulating tau homeostasis relevant to Alzheimer’s disease and related tauopathies.

## Introduction

Alzheimer’s disease (AD) and associated tauopathies are characterized by the accumulation of tau in neurons, resulting in the formation of neurofibrillary tangles, neuronal death, and brain atrophy. In healthy control brains, tau (gene name: *MAPT*), a microtubule-associated protein, is known to aid in microtubule stabilization and in supporting cytoskeletal structure. In pathological conditions, tau becomes hyperphosphorylated and detaches from microtubules, leading to microtubule destabilization. Following microtubule detachment, tau aggregates, forming neurofibrillary tangles characteristic of AD and other tauopathies [20, 30].

Proteostatic failure is a key contributor to neuronal dysfunction and degeneration in aging and age-related diseases [18, 30]. Proteostasis is necessary to maintain proper protein balance in healthy cells; it regulates proteins from synthesis, to folding, trafficking, and degradation. While proteostasis is important for many cell types, it is essential for post-mitotic, long-lived neurons that cannot undergo self-renewal and require mechanisms to dispose of unwanted cellular waste. Loss of proteostatic regulation leads to an accumulation of damaged proteins, organelles, and misfolded proteins, contributing to cell death that is characteristic of neurodegeneration.

Neurons primarily rely on two forms of proteostatic degradation systems: the ubiquitin-proteasome system (UPS) and autophagy [1, 14]. The UPS is responsible for the rapid turnover of soluble, short-lived proteins; autophagy targets larger substrates such as long-lived proteins, protein aggregates, and organelles [22, 23]. Both mechanisms have been shown to degrade tau, and in AD brains, pathological tau is associated with subcomponents of the UPS and autophagy[16, 18, 28]. Unlike aging brains, which have been shown to have decreased proteostatic regulation over time, both the UPS and autophagy have high levels of activity in the developing brain and are essential for proper neuronal development [9, 17, 30].

While tau deposition is characteristic of neurodegenerative tauopathies in the aging brain, we have shown that the developing human brain expresses high levels of tau, similar to what is seen in AD. In contrast to the toxicity seen in age-related neurodegenerative disease, high levels of tau in the developing brain are not associated with adverse consequences. In fact, additional investigations revealed that increased tau expression in the developing human brain, which appears as early as the second trimester, accompanies and can serve as a marker of neuron maturation [12, 13]. However, the mechanisms behind the remarkable resilience of the developing human brain to elevated tau and the absence of tau toxicity remain to be elucidated. In this study, we set out to determine the role of the UPS and autophagy in protecting developing neurons from tau toxicity and whether loss of protein degradation mechanisms in developing neurons would create a model of tau-induced toxicity and cellular death.

## Materials and Methods

### Tau Interactome Data Acquisition and Processing.

Tau-interacting proteins were obtained from previously published co-immunoprecipitation and mass spectrometry data collected in our lab [6]. Briefly, peptides were analyzed by LC–MS/MS using a QExactive HF mass spectrometer and searched with MaxQuant/Andromeda (FDR 1% at peptide and protein levels) followed by Perseus normalization and filtering, as described [6]. For the current analysis, we used the list of identified proteins. To reduce noise, we required that proteins have valid intensity values in at least three replicates within a given condition (fetal, adult control, or AD). Proteins not meeting this threshold were excluded from that condition. Protein intensities represent log2-transformed LFQ intensities normalized per sample as described in Betters et al., 2023. Gene symbols for retained proteins were used as input for GO Biological Process enrichment. Fisher’s exact test was applied for significance testing, and results were corrected for multiple comparisons using the False Discovery Rate (FDR) method (adjusted p < 0.05).

### Tissue Procurement.

Formalin-fixed paraffin-embedded (FFPE) tissue was obtained from the Iowa Neuropathology Resource Laboratory (INRL). Individual cases used, demographic information, and sources are listed in **Table 1**. Each case was reviewed by an experienced neuropathologist (MMH) to ensure that it contained appropriate sections of cerebral cortex. The University of Iowa’s Institutional Review Board determined that, since this project exclusively used tissue from deceased individuals, it does not constitute human subjects research under the NIH Common Rule (determination #201706772). All methods were conducted in accordance with the relevant laws, regulations, guidelines, and ethical standards of our institution, with the 1964 Helsinki declaration and with its later amendments or comparable ethical standards [27].

### Immunofluorescence (IF).

Immunofluorescence was performed on formalin-fixed, paraffin-embedded (FFPE) tissue. Briefly, 5 μm FFPE sections were baked for one hour at 55 °C. Deparaffinization and tissue rehydration steps include washing in xylene (2 × 5 min), 100% ethanol (2 × 5 min), 90% ethanol (5 min), 70% ethanol (5 min), and milliQ H_2_O (5 min). Antigen retrieval was performed in antigen retrieval solution (10 mM sodium citrate, 0.084% Triton X-100, MilliQ H_2_O) at 90 °C for 10 minutes. Slides were washed in 1X PBS and blocked in blocking buffer [3% bovine serum albumin (BSA), 0.36% Triton X-100, and 1X PBS] for 1 hour at room temperature. Primary and secondary antibodies were diluted according to the manufacturer’s guidelines, with the dilutions listed in **Table 2** and **Table 3**. Primary antibody incubation was performed overnight at 4°C, and secondary antibody incubation was performed for 1 hour at room temperature, protected from light. We used TrueBlack Lipofuscin Autofluorescence Quencher diluted according to the manufacturer’s guidelines (23007, Biotium; Sam Fransico, CA, USA) for 30 seconds to quench autofluorescence. To mount the slides, we used VECTASHIELD PLUS Antifade Mounting Medium with DAPI (H-2000; VectorLabs; Neward, CA, USA). No primary controls were used to ensure the specificity of antibody staining.

### Confocal Imaging and Image Analysis.

Prepared IF samples were imaged using a Leica DMI8 inverted confocal microscope using LASX and a HC PL APO CS2 100x/1.40 oil objective; pinhole ~1AU for all channels (LASX auto) and unchanged across acquisitions. Excitation: 405 nm (DAPI), 488 nm (Alexa Fluor 488), 552 nm (Alexa Fluor 555), 638 (Alexa Flour 647). Emission detection windows: 502–557 nm (AF488), 557–643 nm (AF555), 643–789 nm (AF647); DAPI collected with the system’s UV optics on HyD/PMT detectors with sequential line scanning to avoid bleed-through. Laser power and detector gain were adjusted to place the signal within the linear dynamic range, while avoiding saturation (confirmed by the LUT “over/under” display), and remained constant between samples. Images were acquired with a voxel size of 0.114 × 0.114 × 0.297 mm (LAS X auto); acquisition settings were held constant across conditions. For figure preparation, panels were created in FIJI (ImageJ2 v2.16.0/1.54p). A single representative z-plane per field with a fixed-size ROI was applied to generate orthogonal x-z/y-z views. A merged composite image of all channels from the same z-plane/ROI was generated for each sample. Maximum-intensity projections of the full stacks were also generated, with the ROI location indicated. Only global linear contrast adjustments were used with identical ranges across samples; no nonlinear processing was applied. Scale bars were rendered from image metadata.

### Stem cell differentiation.

HumaniPSC-derived neuronal progenitor cells (NPC) from control donors were obtained from NeuraCell (Albany, NY) (cell line F12442.5). The cell line was searched on ICLAC’s Register of Misidentified Cell Lines and was not listed. The parent stem cells were characterized for pluripotency and differentiated into NPCs by Neuracell^19^. NPCs were cultured using STEMDiff Neural Progenitor Medium (STEMCELL Technologies, Cat. No. 05833) according to the manufacturer’s protocols. Differentiation into neurons was carried out using the STEMDiff Forebrain Neuron Kit (STEMCELL Technologies, Cat. No. 08600) and the STEMDiff Neuron Maturation Kit (STEMCELL Technologies, Cat. No. 08605) according to the manufacturer’s protocols, and successful differentiation was verified by immunocytochemistry. Matrigel was diluted according to lot-specific dilution guidelines provided by Corning in 25 mL of Dulbecco’s Modified Eagle Medium (DMEM) with F-12 and 15 mM HEPES Buffers (DMEM/F-12 Buffer with 15 mM HEPES: STEMCELL Technologies, Cat. No.36254). Plates were coated with diluted Matrigel and incubated at 37°C for 1 hour before cell plating.

### Cell Treatments.

Mature forebrain neurons were treated with the drugs at concentrations outlined in **Table 4**. The treatment concentrations of epoxomicin, TCH165, and bortezomib (proteasome activators and inhibitors) were determined by treating mature neurons with a dose-response curve of the specific compound and performing western blotting on cell lysates following 24 hours of treatment to observe changes in protein ubiquitination (**Fig. S2**). Treatment concentrations and incubation periods for rapamycin, starvation, and bafilomycin were determined and modified based on prior studies primarily based in neurons [21, 32, 33, 39, 46]. Experimental treatment periods ranged from 24 to 96 hours, depending on the type of experiment. Cells undergoing starvation treatments during the live-cell imaging experiments were treated with media containing ¼ Brainphys^™^ Imaging Optimized Medium (STEMCELL Technologies, Cat. No. 05796) and ¾ HBSS with 10 mM HEPES, without phenol red (STEMCELL Technologies, Cat. No. 37150). The untreated control cells were incubated in Brainphys^™^ Imaging Optimized Medium (STEMCELL Technologies, Cat. No. 05796) with neuron supplements from the StemDiff Neuron Maturation Kit (STEMCELL Technologies, Cat. No. 08605) according to the manufacturer’s protocols. Cells undergoing starvation treatments before lysing for western blotting were treated with ¾ HBSS with 10 mM HEPES, without phenol red (STEMCELL Technologies, Cat. No. 37150) and ¼ Brainphys^™^ Neuronal Medium from the StemDiff Neuron Maturation Kit (STEMCELL Technologies, Cat. No. 05796) without the added supplements. Like the treatment media, control media did not include phenol red.

### Immunocytochemistry.

All primary and secondary antibodies used in this proposal, along with their concentrations, are listed in **Tables 2** and **3**. No primary controls were used to ensure the specificity of the antibody staining. Following the required maturation and treatment period, the cells were washed once with 1X phosphate-buffered saline (PBS) before fixation for 30 minutes with 10% formalin at room temperature. Following fixation, the cells were washed twice with 1X PBS and stored in 1X PBS with 0.01% sodium azide at 4°C. To remove the storage buffer, the cells were washed 3 times for 1 minute each with 1X PBS. For membrane permeabilization, the cells were incubated in extraction solution (0.02% Triton-X 100 in 1X PBS) (Fisher Scientific, Cat. No. BP151) for 10 minutes at room temperature. The fixed cells were then blocked in blocking solution (10% heat-inactivated fetal bovine serum (hiFBS) in 1x PBS) (R&D Systems, Cat. No. S1245914) for 30 minutes at room temperature. The primary antibodies were diluted in serum solution (1% hiFBS in 1X PBS) according to the dilutions stated in **Table 2**. The cells were incubated in the primary antibody dilution overnight at 4°C on a rotator shaker (setting: low speed). Following primary antibody incubation, the samples were washed five times for 1 minute each with 1X PBS. The secondary antibodies were diluted in serum solution according to the dilution ratios stated in **Table 3**. The cells were incubated in the dark at room temperature for 1 hour. After incubation in the secondary antibody dilution, the samples were washed 5 times for 1 minute each. NucBlue^™^ Fixed Cell ReadyProbes^™^ Reagent (1 drop/1 mL) (DAPI) was added to each well and incubated in the dark at room temperature for 10 minutes (Invitrogen, Cat. No. R37606). The samples were washed 3 times for 1 minute each and imaged.

### Image Acquisition & Figure Creation.

Image acquisition was performed using a Cytation5 (Agilent, Cat. No. BTCYT5PW) with the GEN5PRIME software (Agilent, Cat. No. BTGEN5IPRIM). Validation imaging was conducted at 20X (Cat. No. BT1320517, Agilent) using a combination of the DAPI filter cube (Agilent, Cube: Cat. No. BT1225100; LED: Cat. No. BT1225007), GFP filter cube (Agilent, Cube: Cat. No. BT1225101; LED: Cat. No. BT1225001), the TRITC filter cube (Agilent, Cube: Cat. No. BT1225125; LED: Cat. No. BT1225012), and/or the CY5 filter cube (Agilent, Cube: Cat. No. BT1225105; LED Cat. No. BT1225005) as necessary. Microscopic fields were chosen randomly, and the acquisition settings and image processing parameters remained consistent across all images/wells. Imaging was performed blinded to experimental conditions to prevent bias. Figure images were created with the GEN5PRIME software and Adobe Illustrator.

### LAMP2A Image Analysis.

After treating mature human forebrain neurons with bafilomycin and starvation for 24 hours (see the cell treatments section and **Table 4** for treatment concentrations), immunocytochemistry and image acquisition were performed as detailed above. Treatment conditions were cultured in triplicate, and triplicate images were taken per well. Following image acquisition, ImageJ was utilized to process the raw photos. All images were converted to 8-bit images, and the LAMP2 and DAPI channels were thresholded with cutoffs of 88/255 and 50/255, respectively. Particle analysis was performed on the thresholded LAMP2 images to obtain the total area (pixels^2^) localized to the soma and on the thresholded DAPI images for total cell count. The total area of LAMP2A staining localized to the soma was normalized to the total DAPI count. Following normalization to DAPI, p-values were obtained utilizing unpaired two-tailed Welch’s t-tests comparing treated cells to vehicle-treated cells (control). All image analysis was performed with ImageJ [35]. Data analysis was conducted using GraphPad Prism (version 10.4.1).

### Creating Fluorescent 1N4R eGFP Tau Lentivirus.

pLJM1 EGFP-tau (Addgene, Cat. No. 108868) was streaked for single colonies from an agar stab and incubated for 12–18 hours at 37°C. Following overnight incubation, two to three colonies were inoculated in 5 mL of LB culture medium (25 grams of Luria Broth (Cat. No. L24400–500.0, RPI), 1 mL ultrapure H20, and 100 mg of ampicillin (RPI, Cat. No. A400040–5.0) and incubated on a rotary shaker at 225 rpm for 5 hours at 37°C. Plasmid purification was performed using the ZymoPURE II Plasmid Maxiprep Kit (Zymo Research, Cat. No. D4202). Following plasmid purification, 300 μg of the plasmid DNA was sent to the University of Iowa Viral Vector Core for sequencing and lentiviral particle production. Plasmid encapsulation into third-generation HIV-based lentiviral particles was conducted using the Invitrogen Corporation ViraPower^™^ Lentivirus Expression System (Invitrogen, Cat. No. K4975–00). The core facility measured viral titers and MISSION/ pLKO.1-puro-CMV-TurboGFP Positive Control transduction particles (Sigma-Aldrich, Cat. No. SHC003V) were utilized to determine the optimal multiplicity of infection (MOI). The UI Core conducted both microscopy and flow cytometry to assess transfection efficiency.

### Generating Stable 1N4R eGFP Tau-Expressing Neurons.

F12442.5 Neural Progenitor Cells (NPCs), purchased from Neuracell, were seeded at a density of 1.0 × 10^6^ cells per well on Matrigel-coated 6-well Corning plates. The cells were plated, differentiated, and matured as described above. Approximately 16–24 hours following plating, the NPCs were transduced with the lentiviral particles (eGFP Tau – MOI 25 | titer 7.9×10^8^ TU/mL). 24 hours post-transfection, the lentiviral-containing media was removed, and fresh media was added. Seventy-two hours post-transfection, puromycin selection (Gibco, Cat. No. A1113803) at a concentration of 1 μg / mL was conducted for 3 – 5 days. In addition to puromycin selection, validation of successful transduction was also performed via microscopy with the Cytation5 to assess eGFP-tau expression. Seven days post-plating, the transduced NPCs were passaged and plated to undergo differentiation and maturation into forebrain neurons utilizing STEMCELL Technologies’ kits and protocols described above. Throughout the differentiation process, eGFP-tau expression was monitored regularly using the Cytation5 to ensure consistent expression and healthy cell morphology.

### Live/Dead Assay.

Mature forebrain neurons were treated with epoxomicin, bortezomib, TCH-165, and bafilomycin (concentrations outlined in **Table 4**) for 24, 48, and 72 hours. Following incubation, the treated neurons were incubated with Invitrogen’s Live/Dead Cell Imaging Kit (488/570) (Invitrogen, Cat. No. R37601) following the manufacturer’s guidelines and imaged with the Cytation5.

### Whole-Cell Patch Clamp Recording.

Cover-slips containing cultured neurons were transferred to a submerged recording chamber and perfused with aCSF (32–34°C, 95% O_2_ / 5% CO_2_). Drugs were not perfused during the recording. Micropipettes were filled with a potassium-based intracellular solution containing (in mM) K-gluconate (120), EGTA (0.15), HEPES (10), KCl (20), Na-GTP (0.3), Mg-ATP (4), phosphocreatine (10, pH=7.2, 289 mOsm). Neurons were identified using IR-Dodt-Gradient Contrast (BX51WIF, 40x water immersion objective, Olympus with an infracontrast DGC, Luigs & Neumann), imaged with a CS505MU CMOS camera (Thorlabs) and recorded with an integrated patch-clamp amplifier (Double-IPA, Sutter Instruments). In current clamp mode, hyperpolarizing and depolarizing currents ranging from −100 pA to 300 pA (20 pA steps) were injected, and the resulting voltage changes were recorded. For a more precise rheobase determination, we injected currents from −40 pA to 80 pA in 5 pA steps. Series resistance and whole-cell capacitance were estimated and compensated. Recordings were discontinued if the series resistance increased by >25% during the experiment. Data was acquired at 10 kHz and low-pass filtered at 3 kHz and analyzed post-acquisition using SutterPatch, IgorPro9, and GraphPad Prism (version 10.4.1). Unpaired t-tests were utilized to determine the difference in resting membrane potential, capacitance, and input resistance between young and old neurons (n=15).

### Proteasome Activity Assay.

Cultured neurons were washed twice with cold 1X PBS with 1:100 HALT Protease Inhibitor Cocktail (Thermo Fisher Scientific, Cat. No. 78441). The cells were gently scraped from their wells in a small amount of cold 1X PBS with HALT. The cells were spun down at 4°C at 300 × g for 5 minutes. The pellet was resuspended in homogenization buffer [50 mM Tris Reagent (Invitrogen, Cat. No. 2769802), 5 mM MgCl_2_, 250 mM sucrose, 1 mM DTT, 2 mM ATP, and 1:100 HALT Phosphatase & Protease Inhibitor] and sonicated in a QSonica sonicator at 4°C for 10 min (10 second pulses with 30 second pauses at 30% amp). 10 μg of homogenized protein samples per sample was added to 150 microliters of incubation buffer [50 mM Tris Reagent, 40 mM KCL, 5 mM of MgCl_2_, 1:100 HALT, 1 mM ATP, 1 mM DTT, 0.05 mg/mL Bovine Serum Albumin, and 20 μM Suc-LLVY-AMC (AAT Bioquest, Cat. No. 13453]. This was done in triplicate. Each triplicate sample was incubated in a glass-bottom 96-well plate (Cellvis, Cat. No. P96–0-N) for 1 hour, protected from light at 37 °C. A Bio-Tek plate reader was used to measure proteasome activity, measuring fluorescence activity (ex380 nm and em460 nm). Data analysis was performed with GraphPad Prism (version 10.4.1), utilizing ANOVA with the Brown-Forsythe correction.

### Western blotting.

Western blot antibody validation was performed prior to blotting experimental samples. HT7 (Invitrogen, MN1000) was validated using the appropriate positive and negative controls from human brain samples as previously described [5]. LC3B, P62, and P4D1 were previously validated by the manufacturer. Cells were washed twice with 1X and then scraped in cold 1X PBS with HALT Protease Inhibitor Cocktail at a concentration of 1:100 (Thermo Fisher Scientific, Cat. No. 78441) on ice. Cells were spun at 300 × g for 5 minutes at 4 °C. The supernatant was removed, and the cells were flash frozen in liquid nitrogen for 5 minutes to lyse them. Following flash freezing, the samples were thawed on ice in a small volume (<100 μl) of 3X homogenization buffer [137 mM NaCl, 20 mM Tris-HCL, 10% glycerol, 1% Triton-X, 1:100 halt inhibitor cocktail (Thermo Fisher Scientific, Cat. No. 78441), pH to 8] for ~30 minutes with occasional vortexing. After thawing, the samples were passed through a P200 tip 8–10 times to further lyse the cells. The samples were then centrifuged at 15,000 × g for 10 minutes at 4 °C. Following centrifugation, the supernatant was collected, and a Bicinchoninic Acid (BCA) assay was performed to determine the protein concentration. The Pierce Protein Assay Kit (Thermo Fisher Scientific, Cat. No. 23227) was used according to the manufacturer’s protocol. Samples were prepared for western blotting following previously published procedures [5]. 10–30 μg of protein per sample was loaded into 10% Mini-PROTEAN TGX Precast Protein Gels (Bio-Rad, Cat. No. 4561034). The samples were run on the gel at 150V for 60 minutes. The proteins were then transferred utilizing Trans-Blot Turbo PVDF Transfer Packs Mini, 0.2 μm (Bio-Rad, Cat. No. 1704156) with the Trans-Blot Turbo Transfer System (Bio-Rad, Cat. No. 1704150) with the standard Bio-Rad “MIXED MW” setting. Due to the low molecular weight of LC3B and the need to distinguish two bands with similar molecular weights, the LC3B western blot was run on a NUPAGE 4–12% Bis-Tris gel with 1X NuPAGE MES SDS running buffer (Invitrogen, Cat. No. NP002) at 120 volts for 90 minutes. The blot was then transferred onto a 0.2 μm immuno-Blot PVDF membrane (Bio-Rad, Cat. No. 1620177) using the semi-dry transfer method at 120V for 80 minutes [36].

Following blot transfer, the membranes were blocked in EveryBlot Blocking Buffer (Bio-Rad, Cat. No. 12010020) for 5 minutes at room temperature. After blocking, the membranes were placed directly in primary antibody incubation buffer, which consisted of the primary antibody (see **Table 2** for antibody dilution) in EveryBlot Blocking Buffer, for 1 hour at room temperature with gentle rocking. The membranes were then washed 5 times for 5 minutes each with 1% TBST Wash Buffer [0.1% Tween20 & 1X TBS (10x TBS – Bio-Rad, Cat. No. 1706435) diluted in MilliQ water]. Secondary antibodies (see **Table 3**) were diluted in EveryBlot Blocking Buffer, and membranes were incubated with gentle rocking for 1 hour at room temperature. The wash steps were repeated and followed by incubation of the membranes in Clarity Western ECL Substrate (Bio-Rad, Cat. No. 1705060), which was prepared following the manufacturer’s guidelines, for 5 minutes. Imaging was performed with the Bio-Rad ChemiDoc Imager.

ImageJ was used to analyze band intensity [35]. Western blot images were converted into 8-bit images. Image colors were inverted, and a box was drawn around each band to measure band intensity – the box remained the same size regardless of changes in band shape. A measurement of the background was taken with the same measurement box to subtract the band intensity from background intensity. Using GraphPad Prism (version 10.4.1), multiple unpaired two-tailed t-tests between groups with equal variance were utilized to analyze the difference in band intensity.

### qPCR.

Following treatment with epoxomicin, bortezomib, or bafilomycin for 24 hours (**Table 4**), the treated cells were washed twice with ice-cold 1x PBS, and RNA isolation was conducted with the Ambion Purelink RNA Minikit (Invitrogen, Cat. No. 12183018A) following the manufacturer’s guidelines. After successful RNA isolation, RNA concentrations were measured with the Nanodrop. cDNA was synthesized at 50 – 100 ng/μl with the Verso cDNA Synthesis Kit (Thermo Scientific, Cat. No. AB1453A) following the manufacturer’s guidelines. qPCR was performed utilizing Applied Biosystem’s TaqMan Fast Advanced Mastermix (Thermo Fisher Scientific, Cat. No. 4444556) following the manufacturer’s guidelines. Experimental setup included three biological replicates each run in triplicate. Taqman Probes used: HS00902194_m1mapt (MAPT) and GAPDH VIC – HS02786624_q1 (GAPDH). The technical replicates CT were averaged to obtain a single value for each biological replicate. Relative gene expression analysis was performed utilizing 2^−Δ ΔCT^ method [25, 34]. Multiple t-tests were performed with the Holm-Šídák method to correct for multiple comparisons using GraphPad Prism (version 10.4.1).

### Live-cell imaging.

Live-cell imaging was performed with the Agilent Technologies’ Cytation5 (Cat. No. BTCYT5PW) with the GEN5PRIME software (Agilent, Cat. No. BTGEN5IPRIM). Imaging was performed over 96 hours at 37 °C and 5% CO_2_ (2% gradient). Images were taken at 20X magnification with the laser autofocus cube (Cat. No. BT1225010) and the GFP filter cube (Cat. No. BT1225101). Each live-cell imaging experiment utilized three biological replicates per condition. Images were taken hourly in a 5×5 montage per well. GFP analysis was performed by measuring total fluorescence intensity per well with the GEN5PRIME software. Data organization and analysis were performed with RStudio (version 4.4.3) [38] utilizing the following packages: tidyverse [42], lme4 [10] nlme [31], dplyr [43], tidyr [45], data.table [4], reshape2 [41], ggplot2 [40], marginaleffects [2], and svglite [44]. Live-cell analysis code is found in **Supplemental Information S10**.

### RNA sequencing.

The experimental setup included three biological replicates per condition. After treating the cells with epoxomicin (**Table 4**) and the control cells with vehicle for 24 hours, the cells were washed twice with cold 1X PBS on ice. The ThermoFisher Purelink RNA Minikit (Thermo Fisher, Cat. No.12183020) was used to isolate RNA from the pelleted cells. Gene expression profiling using RNA-Seq was performed by the University of Iowa Genomics Division using manufacturer recommended protocols. Briefly, 500 ng of DNase I-treated total RNA was used to prepare sequencing libraries using the Illumina TruSeq stranded mRNA library preparation kit (Illumina, Cat No. RS-122–2101). The molar concentrations of the resulting indexed libraries were measured using the Agilent 2100 Bioanalyzer (Agilent Technologies, Santa Clara, CA) and combined in equal amounts into a pool for sequencing. The concentration of the library pool was determined using the Illumina Library Quantification Kit (KAPA Biosystems, Wilmington, MA) and sequenced using the 100 bp paired-end run configuration on the Illumina NovaSeq 6000 genome sequencer running v1.5 SBS chemistry to generate at least 30M sequence reads per sample — equal aliquots of each sample were run on two lanes. The resulting fastq files were then aligned to the human hg38 reference genome using the STAR aligner, quantified using subreads, and differential expression; reads from the two lanes were summed and analysis carried out using DESeq2, using default independent filtering and Cook’s distance outlier detection, in RStudio as described in previous studies [11, 37]. Volcano plots were generated in R (ggplot2.x;ggrepel v0.9.x) using per-gene log2 fold-change (x-axis) and −log10 (adjusted p) (y-axis; Benjamini-Hochberg). Unless stated otherwise, significance thresholds were |log2FC| ≥ 0.5 and FDR < 0.05; threshold lines were drawn accordingly.

### Hierarchical clustering.

Differentially expressed genes by DESeq2 (FDR <0.05) with |log2FC| > 0.5 were retained. Variance-stabilized counts (VST; blind = FALSE) were z-scored per gene and clustered unsupervised using Euclidean distance and complete linkage in R (hclust). The dendrogram was cut into k = 6 clusters, a value selected from the tree structure before downstream enrichment and held constant. The *MAPT*-containing cluster (cluster 4) and the cluster containing the most upregulated genes (cluster 1) were carried forward for promoter motif/enrichment analyses (Hierarchical analysis R code: **Supplemental Information S12**). Full cluster memberships are found in **Sup. Table 3**, full cluster volcano plot and all DEGs heatmap are provided in **Supplementary Information S7 & S8**.

### Gene Ontology analysis.

GO enrichment analysis was conducted on genes *MAPT* cluster, cluster 1, and unclustered genes (|log2FC| ≥ 0.5; p<0.05) using the Gene Ontology Unifying Biology database and the GO Biological Process Complete annotation dataset [3, 7]. Fisher’s exact test was applied for significance testing, and results were corrected for multiple comparisons using the False Discovery Rate (FDR) method (adjusted p < 0.05). The mapped IDs were reviewed to identify processes of interest and those relevant to the study. The unmapped IDs, which contained non-coding RNA and pseudogenes, were disregarded.

### Homer motif enrichment.

Promoter motif enrichment was performed on direction-specific (upregulated and downregulated) differentially expressed genes. For each set, promoter regions were defined as −300 to +50 bp relative to the hg38 TSS using HOMER (annotatePeaks.pl tss hg 38 -size −300, 60 – list), converted to BED, and analyzed with HOMER v5.1 (findMotifsGenome.pl…hg38) using GC/CpG-matched backgrounds. Q-values were Benjamini-Hochberg adjusted; motifs with FDR <0.05 were considered significant. A sensitivity analysis repeated enrichment using a stricter subset (FDR < 0.05 & |log2FC| ≥ 0.5) was also conducted. Only hits classified by HOMER as “known results” were used in downstream data analysis. For cluster 4, additional analysis using a custom background of DE-downregulated genes excluding cluster 4, was utilized to identify motifs enriched from cluster 4.

### HOMER *MAPT Scanning.*

We defined MAPT promoter windows at −300:+50 and −1000:+100 bp relative to the hg38 TSS using annotatePeaks.pl and scanned for specific position weight matrices (PWMs) with findMotifsGenome.pl -find, using motif files from our HOMER results: **E2F1** (motif_down_hg38/knownResults/known119.motif), **ETV1/ETS** (known40.motif), **TCF3/E2A** (motif_cluster4_vsDOWN/knownResults/known2.motif), and **LHX1** (known149.motif).

### ChEA3 transcription-factor enrichment.

Transcription factor enrichment from gene lists (analyzed separately for up- and down-regulated sets and the *MAPT* cluster set) was performed with ChEA3 (accessed September 2025) [19]. We exported integrated mean rank results and used rank as the primary metric because scores are not comparable across libraries. Transcription factors were considered strong if they achieved rank ≤ 100 in at least one library for the corresponding gene set.

### JASPAR motif confirmation (MAPT).

The MAPT promoter (hg39, −1000 to +100 bp relative to the hg38 TSS, sequence exported from UCSC) was scanned against JASPAR CORE (vertebrates, 2024) motifs for candidate transcription factors using TFBS Tools with a relative profile score threshold of ≥0.85 on both strands. JASPAR2022 human CORE matrices were used; for TFs with multiple matrices (e.g., TCF3), the highest-scoring matrix on the *MAPT* sequence (ID: MA0522.3) was reported; results were consistent across alternative TCF3 matrices (**Sup. Table 2**). We recorded the presence/absence and best score per transcription factor. Transcription factors were prioritized when supported by HOMER motif enrichment and/or ChEA3 (specific library rank) and exhibited a *MAPT* promoter match in JASPAR.

### Figure Creation.

All figures were created using Adobe Illustrator 2025 and with Biorender.com.

## Results

### Distinct and shared biological processes among tau-interacting proteins in fetal, adult, and AD brain.

**Figure 1:**
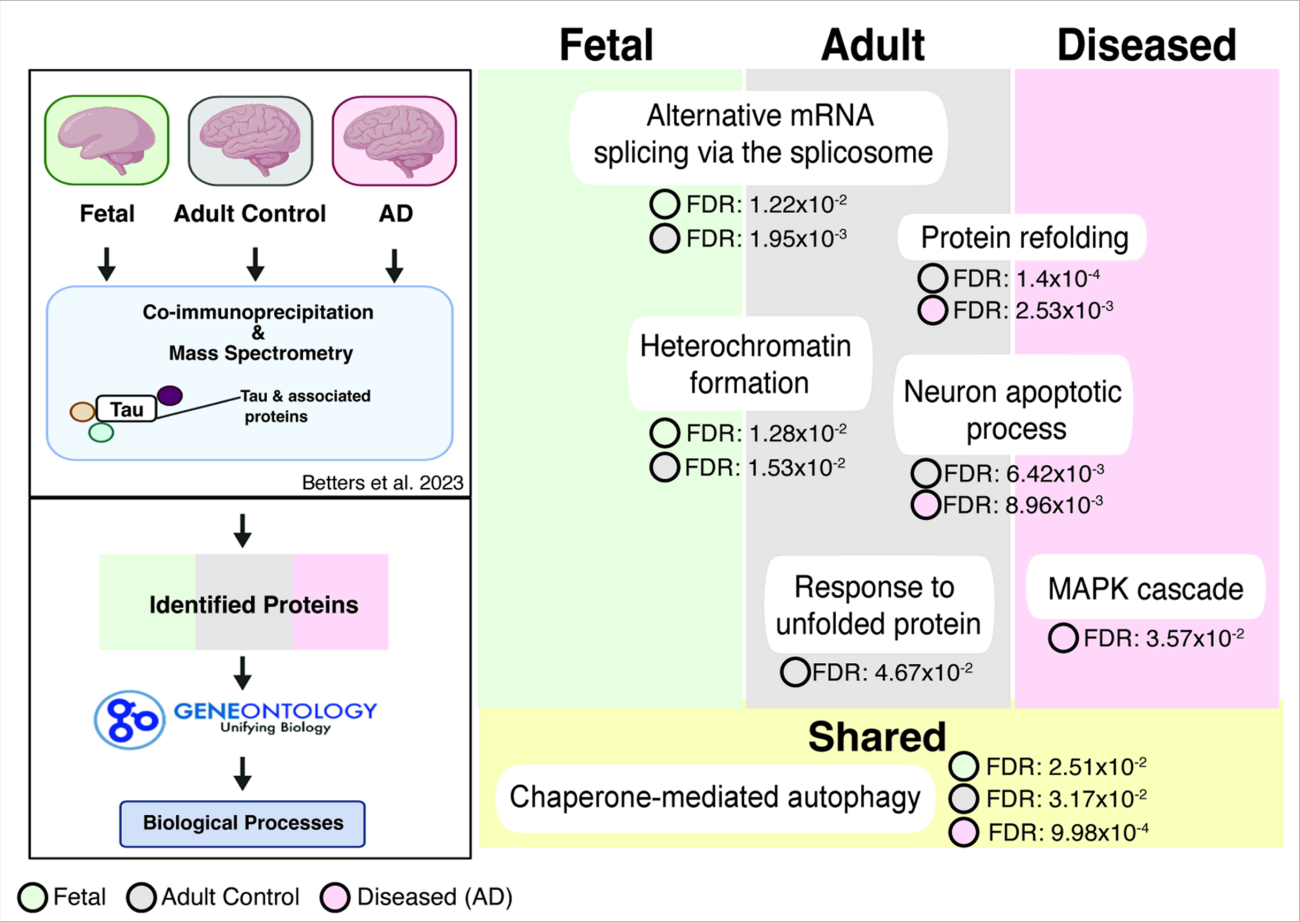
Identified GO Biological Processes of Tau-interacting Proteins from Fetal, Adult Control, and AD brains. Tau-interacting proteins from fetal, adult control, and AD brain co-immunoprecipitated/mass spectrometry datasets were analyzed for enriched GO Biological Process terms. Each dataset was analyzed separately; enrichment used Fisher’s exact test with false discovery rate (FDR) correction for multiple testing; terms with FDR <0.05 are shown. Circle-coded FDR values correspond to the dataset in which enrichment was observed (green = fetal, gray = control, pink = AD). Terms were curated to emphasize processes relevant to tau transcriptional control and proteostasis (full enrichment results, including all terms, effect sizes, and gene sets, in**Sup. table.1**).

We first sought to determine biological processes associated with tau-interacting proteins in fetal, adult control, and diseased human brains. We performed GO enrichment analysis on identified tau-interacting proteins in fetal, adult, and AD brain that was previously in our lab [6]. We observed distinct and shared biological processes in each condition (Fig. 1; All data in **Sup. Table 1**). In the fetal brain and the adult brain, tau interactors were significantly enriched for pathways related to alternative mRNA splicing via the spliceosome (fetal brain FDR: 1.22×10^^−2^, adult brain: 1.95×10^−3^) and heterochromatic formation (fetal brain FDR: 1.28×10^−2^, adult brain: 1.53×10^−2^). In the adult brain specifically, tau interactors also showed enrichment for response to unfolded protein (FDR = 4.67×10^−2^), indicating a reliance on proteostasis-related functions in the adult control brain. In both the adult control brain and AD brain, we observed protein refolding (adult brain FDR: 1.4×10^−4^, AD brain FDR: 2.53×10^−3^) and neuron apoptotic process (adult brain FDR: 6.42×10^−3^, AD brain 8.96×10^−3^). Distinct from the AD brain, we observed tau interactors associated with the MAPK cascade (FDR: 3.57×10^−2^). All three conditions were enriched for chaperone-mediated autophagy (fetal brain FDR: 2.51×10^−2^, adult control brain FDR: 3.17×10^−2^, and AD brain FDR: 9.98×10^−4^). Enrichment of RNA/chromatin and proteostasis-related processes suggested that tau may be regulated at multiple levels, prompting us to examine tau proteostasis in the human brain through the ubiquitin–proteasome system (UPS) and autophagy pathways.

### Representative IF shows localized tau-proteostasis marker association in AD cortex.

**Figure 2:**
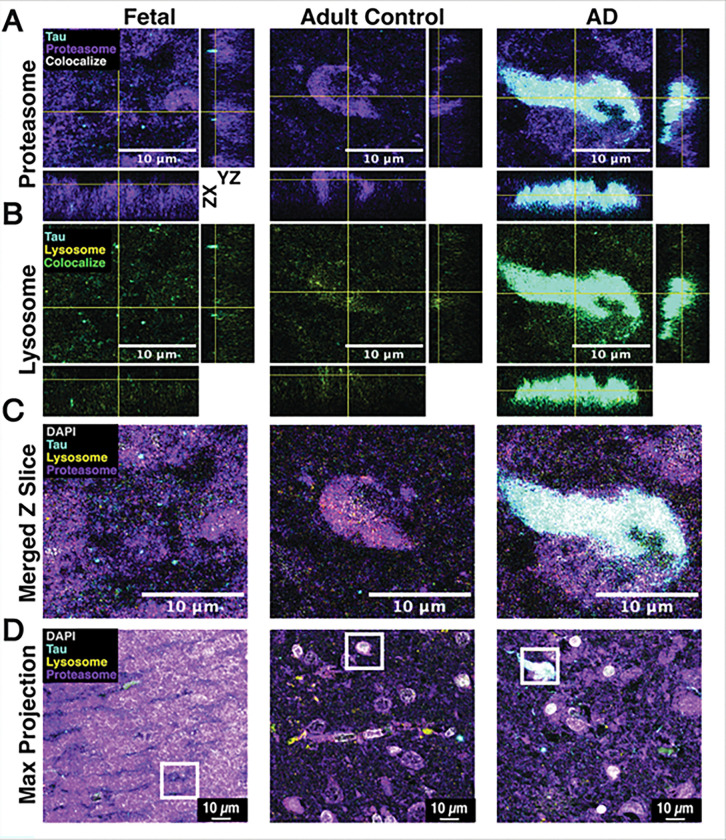
Representative Colocalization of Tau and Proteostasis Markers (PSMA5 & LAMP2A) in Human Fetal, Adult Control, and AD Frontal Cortex (area B1). **A**, Tau (cyan) and PSMA5 proteasome subunit (magenta) with orthogonal x-z/y-z views from a single z-plane ROI per sample in fetal, adult control, and AD brain. **B**, Tau (cyan) and LAMP2A lysosome marker (yellow) with orthogonal views from the same ROI in fetal, adult control, and AD brain. **C**, Merged Z-slice composites (DAPI in grayscale) from the ROIs shown in (A-B). **D**, Maximum-intensity projections of the full stacks with ROI locations indicated (boxes. Scale bars, 10 μM. Images are representative and intended for qualitative illustration; no group-level statistics were performed.

Representative confocal images from fetal (case 1), adult control (case 2), and AD (case 3) frontal cortex (area B(n=1) show local regions where tau signal overlaps with proteasome marker (PSMA5) and lysosome marker (LAMP2A) (**Table. 1**) (Fig. 2). In AD cortex, tau appears nested within proteasome-positive regions (white ROI; orthogonal views), whereas fetal and adult control tissue display minimal apparent overlap. Tau also shows partial overlap with lysosome marker (lamp2a) in AD tissue (orthogonal views), however, most of the tau signal lies outside lysosome-positive pixels. In representative fields, proteasome labeling appears stronger and more extensive in fetal tissues than adult control and AD tissue. Together, these observations led us to probe the mechanisms of tau proteostasis in developing neurons, assessing the consequences of UPS or autophagy disruption.

### Validation of human IPSC-derived neuron maturation.

**Figure 3:**
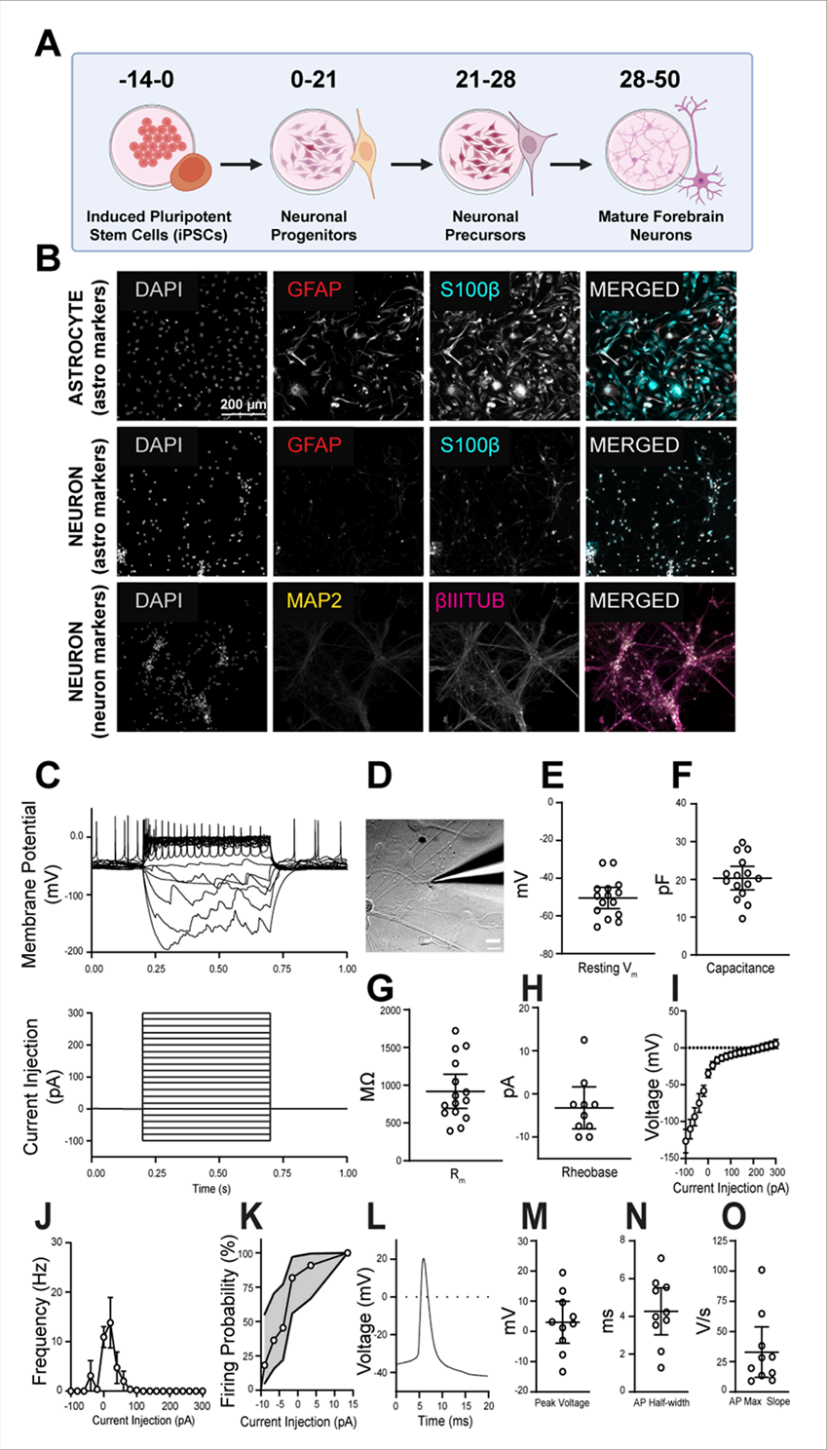
Validation of Forebrain Neuron Maturation. **A**, Schematic showing differentiation of induced pluripotent stem cells (IPSCs) into mature forebrain neurons. **B**, Validation of neuronal differentiation by immunocytochemistry at differentiation (DIF) 43 of neuronal maturation compared to control astrocytes at DIF 66 of maturation. Β-III-tubulin (magenta), GFAP (red), S100β (cyan), and MAP2 (yellow). DAPI in grey. Scale size is 200 mm, which applies to all twelve immunofluorescence images. **C**, Top: Representative voltage responses to current injections. Bottom: Current injection steps (−100 pA to 300 pA, 20 pA steps). **D**, Example of a patched cultured neuron (DIF 50); example image contrast edited with ImageJ. **E**, Resting membrane potential, −50.5 mV [−56.2, −44.9]. **F**, Capacitance, 20.3 pF [17.2, 23.5]. **G**, Membrane resistance, 919 MΩ [692, 1146], n=15. **H**, Rheobase: −3.25 pA, [−8.10, 1.60], n=10. **I**, Current-voltage (IV) curve, slope (Rm) 860 MΩ [828, 892], R^2^=0.9996, p<0.0001. **J**, Action potential frequency-current (FI) curve, mean ± SEM. **K**, Firing probability, mean ± 95% CI (shade). **L**, Representative voltage trace of an action potential. **M**, Peak action potential voltage: 3.03 mV [−3.91, 9.97]. **N**, Action potential half-width, 4.27 ms [3.03, 5.51]. **O**, Action potential maximum slope: 32.9 V/s [12.0, 53.8], n=10. White circles: Individual neurons. Lines: Mean ± 95% CI.

To assess tau in response to proteosome modulation in developing neurons, we cultured forebrain neurons from human neural progenitor cells (NPCs) generated from an adult control induced pluripotent stem cell (iPSC) line. After validating the NPCs (**Fig. S1**), we successfully differentiated the NPCs into forebrain neurons with minimal astrocytes (Fig. 3A–B). These forebrain neurons had intrinsic excitability (Fig. 3C–O). There were no significant differences in resting membrane potential (p=0.86), capacitance (p=0.58), or input resistance (p=0.94) between young (DIF 51–55) and older (DIF 61–62) cultured neurons. This demonstrates that our cells represent immature neurons and are capable of differentiating into electrophysiologically mature pure neuronal cultures. After validating the markers for successful neuronal differentiation with intrinsic excitability and minimal astrocytes, we began to modulate proteostatic regulation.

### Epoxomicin and bortezomib impair proteasome function, while bafilomycin impairs autophagy.

**Figure 4:**
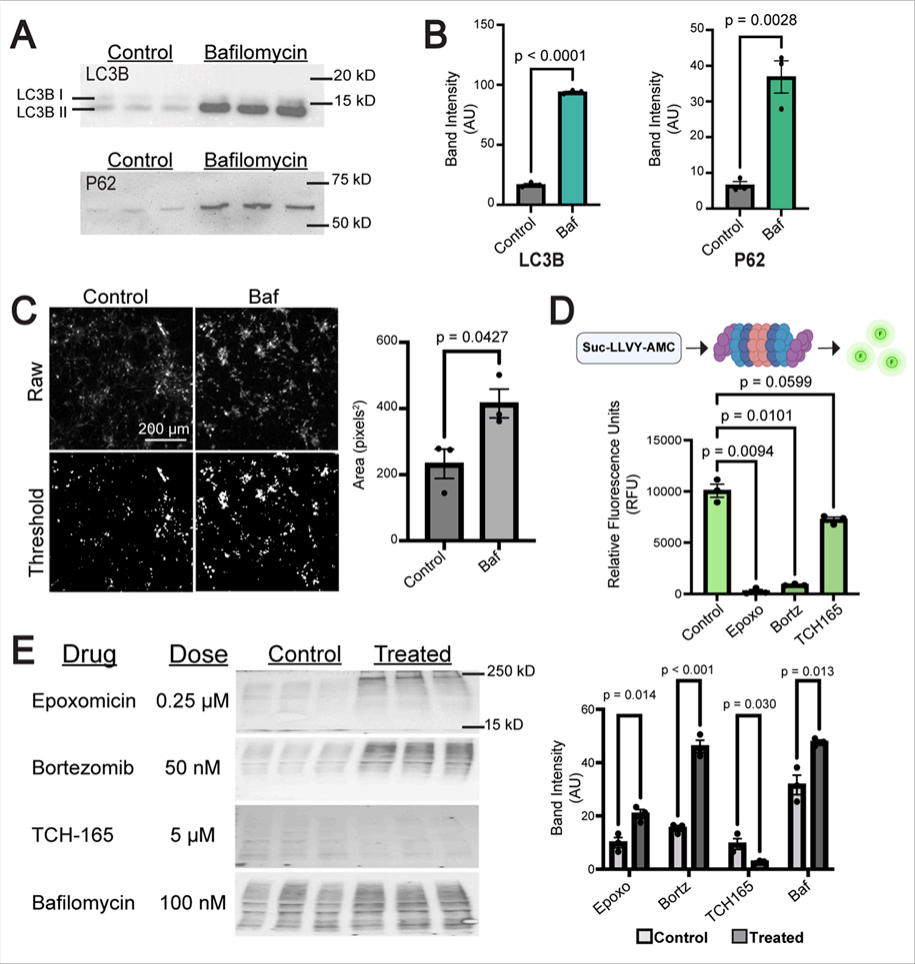
Validation of Key Reagents. **A-B**, Western blots performed with forebrain neuron lysates, probed for LC3B and P62 following treatment with 100 nM of bafilomycin for 24 hours, and analysis of band intensity (AU = arbitrary units) via unpaired two-tailed t-test (Full western blot and amido black: **Fig. S5.E**) **C**, Representative raw immunoFLuorescence images stained for LAMP2A following 24 hours of treatment with 100 nM Bafilomycin vs control vehicle-treated neurons; the thresholded images highlight staining specific to the soma. Analysis of area (pixels^2^) was performed on the thresholded images comparing treatment conditions (control vs. 100 nM Baf) (autophagy activation conditions**: Fig. S4.A**). P-values were derived from unpaired two-tailed Welch’s t-tests. Scale size is 200 mm, which applies to all four immunofluorescence images. **D**, Schematic of the proteasome assay (top). After 48 days of differentiation (DIF 48), neurons were treated with indicated inhibitors for 24 hours, lysed, and proteasome activity measured using SUC-LLVY-AMC fluorogenic indicator. P-values were calculated using Brown-Forsythe and Welch ANOVA. **E,** Western blots were performed on forebrain neuron lysates, probed for ubiquitin (P4D1), with treatments for 24 hours and concentrations as indicated. Analysis of total band intensity was performed, and p-values were calculated with multiple unpaired t-tests (autophagy activation conditions **Fig. S4.B**). Full-size western blots and amido blacks (**Fig. S5.A-B**). All error bars for all statistical tests are +/− SEM.

To perturb UPS and autophagy function in IPSC-derived neurons, we treated differentiated neurons with epoxomicin or bortezomib, both UPS inhibitors, and/or bafilomycin, an autophagy inhibitor. Following treatment with bafilomycin (100 nM), we observed a significant increase in LC3B-II and P62 levels, both of which are proteins associated with autophagosome membranes [39, 40], compared to control cells (p < 0.001 and p = 0.0028, respectively) (Fig. 4A–B), indicating impaired autophagic function. We also examined LAMP2A, a lysosome-associated receptor protein [8]. We saw increased positive LAMP2A staining in the soma of bafilomycin-treated neurons compared to untreated control (p=0.0427), indicating an increase in lysosomes due to inhibition of autophagic flux (Fig. 4C). In response to treatment with the UPS inhibitors, as expected, 0.25 μM epoxomicin and 50 nM of bortezomib, both known UPS inhibitors [26, 47], significantly reduced proteasome function using a fluorescent indicator assay (p=0.0094 and p=0.0101, respectively). Further validation with western blotting indicated that, epoxomicin, bortezomib, and bafilomycin all increased levels of ubiquitinated proteins (p=0.014, p<0.001, and p=0.013, respectively) (Fig. 4D). Live/dead assays following treatment with epoxomicin, bortezomib, and bafilomycin indicated minimal cell death compared to control following 24-, 48-, and 72-hours of treatment incubation **(Fig. S3 A-B)**. These findings indicated successful inhibition the UPS and autophagy with limited cell death at the determined treatment concentrations.

### Developing human neurons have high baseline levels of autophagy and proteasome function.

To assess the effects of increased UPS and autophagic function on tau proteostasis, we assessed the effects of known treatments on our developing neurons, however, we observed no significant increase in UPS or autophagy function in response to treatment. TCH-165, an enhancer of proteasome activity [29], had no significant effect on proteasome function in our neuron cultures but did show a modest decrease in ubiquinated protein levels (p=0.030) (Fig. 4D–E), suggesting a high baseline level of proteasome activity. Rapamycin and starvation, both known enhancers of autophagy, both showed no significant changes, suggesting high baseline levels of autophagy in developing neurons (rapamycin and starvation **Fig. S4 A-B;** full rapamycin and starvation western blots in **Fig. S5.B**). This suggests that immature neurons have high baseline levels of both proteasome activity and autophagy, making these pathways difficult to upregulate in our system.

### Impairment of proteostasis in iPSC-derived neurons reduces tau levels.

**Figure 5:**
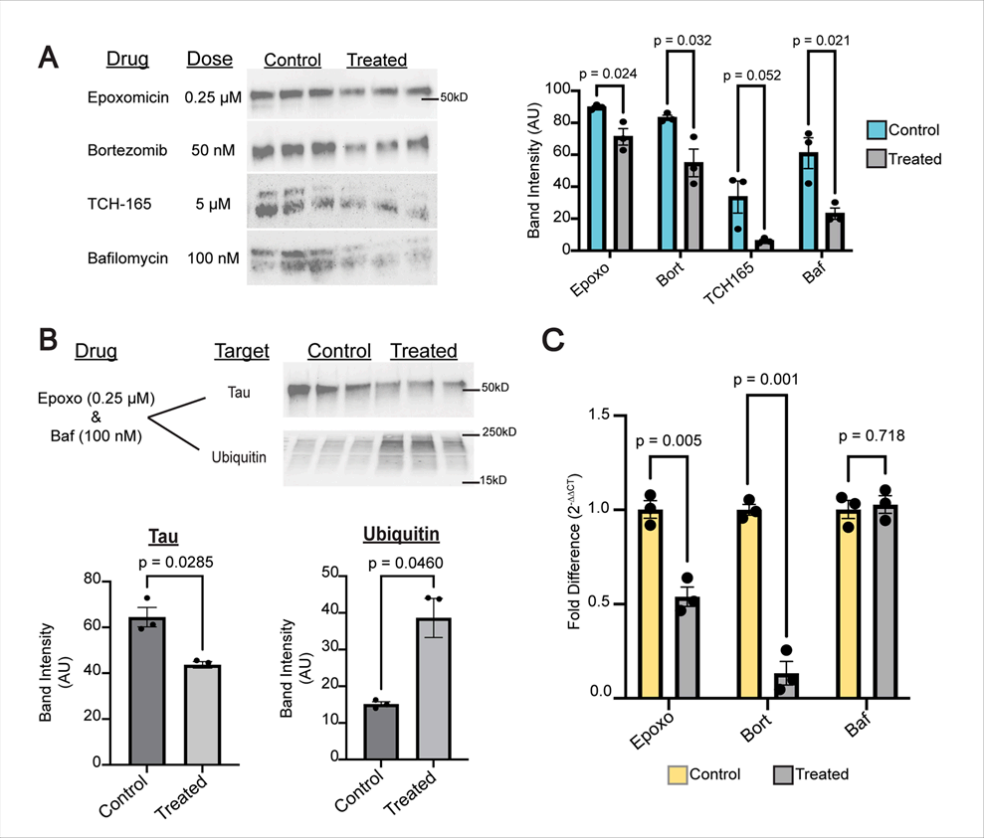
Proteostatic Inhibition in Mature Forebrain Neurons Decreases Total Tau Expression. **A**, Western blots performed on neuron forebrain lysates, probed for tau (HT7) with treatments for 24 hours and concentrations as indicated (left). Analysis of total band intensity; p-values calculated with multiple unpaired t-tests (right) (autophagy activation conditions **Fig. 64.A**) **B,** Western blots and analysis performed on forebrain neurons co-treated for 24 hours with 0.25 μM epoxomicin and 100 nM bafilomycin compared to untreated control probed for tau (HT7) (top) and ubiquitin (P4D1) bottom; p-values obtained with unpaired two-tailed Welch’s t-tests. **C**, Quantitative PCR (qPCR) examining changes in MAPT expression, performed on cDNA from lysed forebrain neurons treated with the validated concentrations (from panel A) for 24 hours; p-values obtained by multiple unpaired t-tests with the Holm-Šídák correction. Error bars for all statistical tests are +/− SEM. Full Western blots and amido blacks found in **Fig. S5.C-D,G**.

Following validation of our modulators of the UPS and autophagy, we sought to assess their effects on tau expression. We first examined changes in tau expression with western blotting (Fig. 5A) (full western blots: **Fig. S5 C-D**). Unexpectedly, we saw that treatment with epoxomicin and bortezomib, both inhibitors of the ubiquitin proteasome system, significantly decreased tau protein levels (Epoxo: p = 0.024, Bort: p=0.032). Treatment with TCH165, a known enhancer of ubiquitin proteasome activity, also reduced tau protein levels compared to vehicle-treated control neurons, with a p-value approaching significance (p = 0.052), and treatment with bafilomycin (autophagy inhibitor) significantly reduced tau levels (p = 0.021) (Fig. 5A). We saw no significant change following treatment with autophagy activators: rapamycin (p = 0.710) or 24 hours starvation (p = 0.172) (**Fig. S6.A;** full rapamycin and starvation western blots in **Fig. S5.D**). After assessing the individual effects of proteasome and autophagy inhibitors on tau, we examined the effects of combined inhibition of the UPS and autophagy with western blotting (Fig. 5B). We saw that combined treatment with 0.25 uM of epoxomicin and 100 nM of bafilomycin significantly decreased tau expression (p = 0.0285) and significantly increased ubiquitin expression (p = 0.0460) compared to vehicle-treated control. These findings show that both individual and combined inhibition of the UPS and autophagy lead to an unexpected decrease in tau protein levels. These findings led us to evaluate the effects of UPS and autophagy perturbation of tau (gene: *MAPT*) at the transcription level.

### UPS inhibition-induced reduction of tau levels is due to reduced MAPT transcription.

After examining decreased tau expression in UPS or autophagy-inhibited neurons, we sought to determine whether the changes in tau expression were occurring at the protein or transcriptional level. To assess this, we performed qPCR on bafilomycin-treated neurons (autophagy inhibitor), epoxomicin-treated neurons (UPS inhibitor), and bortezomib-treated neurons (UPS inhibitor) (Fig. 5C). While we observed no significant changes in *MAPT* expression following 100 nM bafilomycin treatment (p = 0.718), we saw a significant decrease in *MAPT* expression following treatment with 0.25 uM epoxomicin (p = 0.005) and 50 nM bortezomib (p = 0.001). We also observed a significant downregulation in *MAPT* expression in our RNA sequencing data following 24-hour treatment with 0.25 uM epoxomicin (p = 3.33×10^−79^; log_2_FC = −0.883) (Fig. 8). These findings indicate that UPS inhibition affected tau at the transcriptional level, while reduced tau following autophagy inhibition occurs at the protein level.

### Tau expression under a CMV promoter overrides transcriptional regulation.

**Figure 6:**
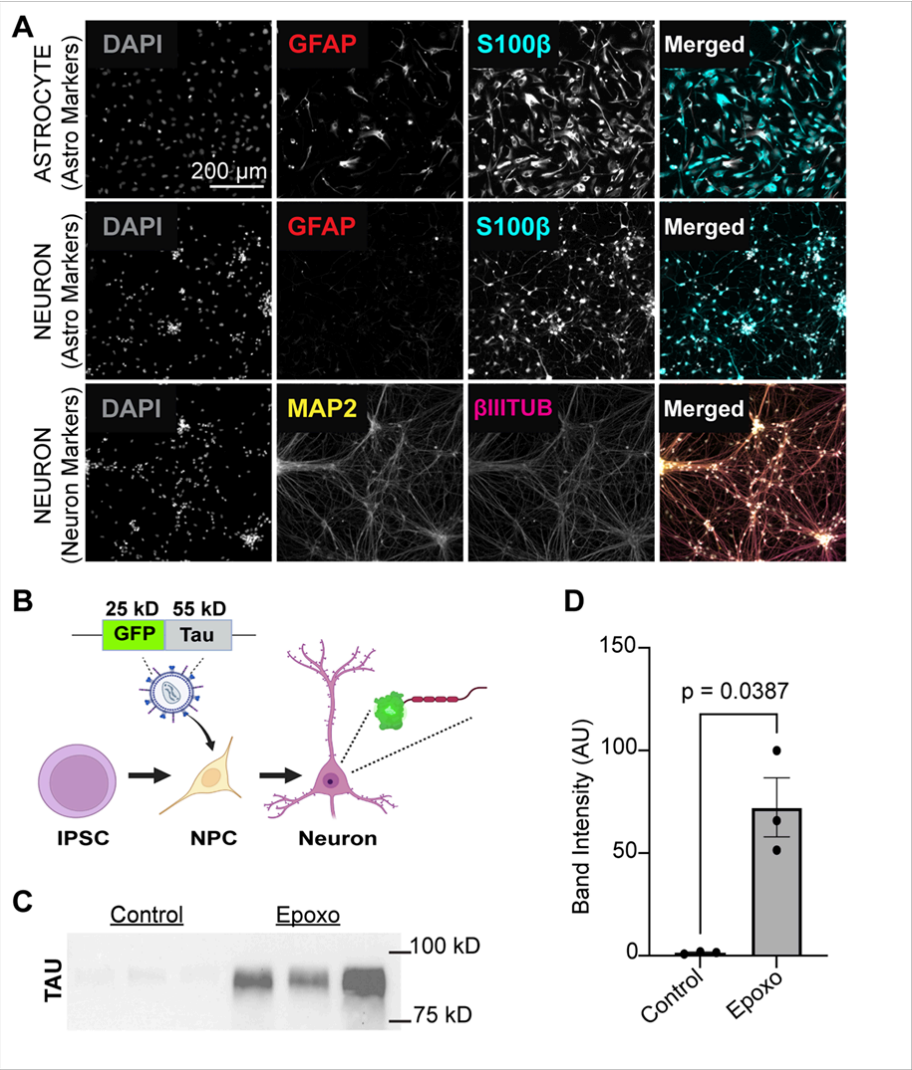
Validation of CMV-Promoted eGFP Tau Forebrain Neurons. **A**, Neuron validation performed on mature eGFp tau neurons (DIF 46) compared to astrocytes control neurons (DIF 66). βIII-tubulin (magenta), GFAP (red), S100β (cyan), and MAP2 (yellow). DAPI in grey. Scale size is 200 mm, which applies to all twelve immunofluorescence images. **B**, Illustration showing transduction of IPSC-derived NPC with eGFP tau lentivirus, which produces ~80 kD tau, and maturation into eGFP tau-expressing neuron **C**, Western blot performed on mature forebrain eGFP tau neurons treated with 0.25 μM epoxomicin for 24 hours compared to untreated control eGFP tau neurons, probed for total tau (HT7). **D**, Statistical analysis performed on western blot band intensity; p-value obtained by unpaired two-tailed Welch’s t-test (right). Error bars are +/− SEM. Full-size western blot and amido black found in **Fig. S5.F**.

**Figure 7:**
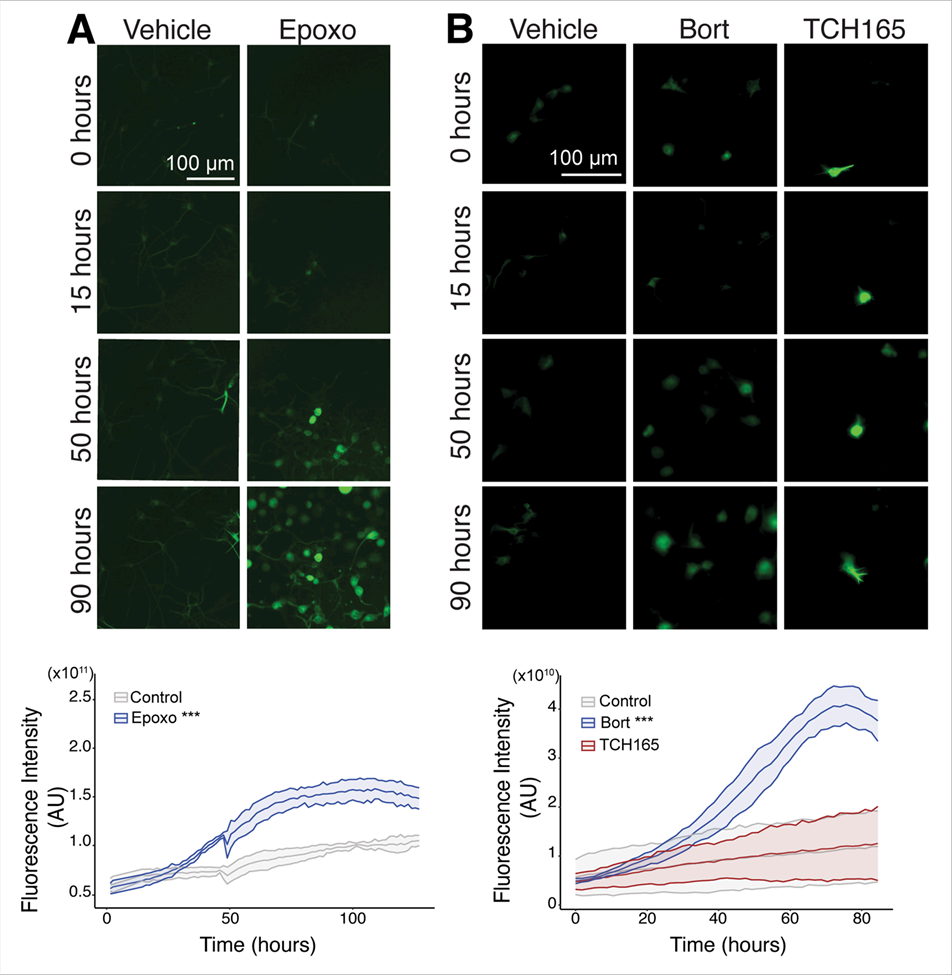
Proteostatic Inhibition in CMV-Promoted eGFP Tau Forebrain Neurons Increases Total Tau Expression. Live-cell imaging performed on eGFP tau neurons. **A-B**, Representative images from live-cell imaging study of neurons individually treated with 0.25 mM epoxomicin (Epoxo), 50 nM of bortezomib (Bort), and 5 mM of TCH165 (TCH165) compared to vehicle-treated control neurons, imaged following initial treatment (time = 0 hours) for four days (time = 96 hours) to examine changes in GFP fluorescence intensity over time. P-values derived from a generalized linear model including time as a covariate and testing the interaction between treatment group and time (Epoxo & Bort: p<2×10^−16^; TCH165: p=0.1837) (Starvation and bafilomycin condition found in **Fig. S6. D-G**). Analysis code is provided in the **Supplemental Information S10.** Scale bar = 100 mm, which applies to all live-cell images. Phase contrast images found in **Fig. S6 B-C**)

To confirm the role of transcriptional regulation of *MAPT* in UPS-inhibited neurons, we stably overexpressed eGFP-linked tau under a CMV promoter and differentiated the resulting stem cell lines into neurons (Fig. 6A–B). In these cells, inhibition of proteasome function with 0.25 μM epoxomicin showed a significant increase in eGFP-tau protein levels (p=0.0387, band at ~75 kDa) by western blotting (Fig. 6C–D; full western blot in **Fig. S5.F**). We then used live-cell imaging to follow eGFP-tau levels over time and saw a significant increase over time with epoxomicin and bortezomib treatment (p< 2 × 10^−16^ for both comparisons) and no significant change following TCH165 treatment (Fig. 7A–B) (phase contrast **Fig. S6.B-C**). We also observed a significant decrease in response to bafilomycin treatment (p< 2 × 10^−16^) and starvation compared to untreated controls (p=9.39 × 10^−10^) (**Fig. S6.D-G**). These findings indicate that forced tau expression followed by individual proteasome inhibition leads to significantly increased tau protein by overriding transcriptional tau regulation.

### Proteasome inhibition in IPSC-derived neurons upregulated stress-related genes and downregulated microtubule-associated genes.

**Figure 8:**
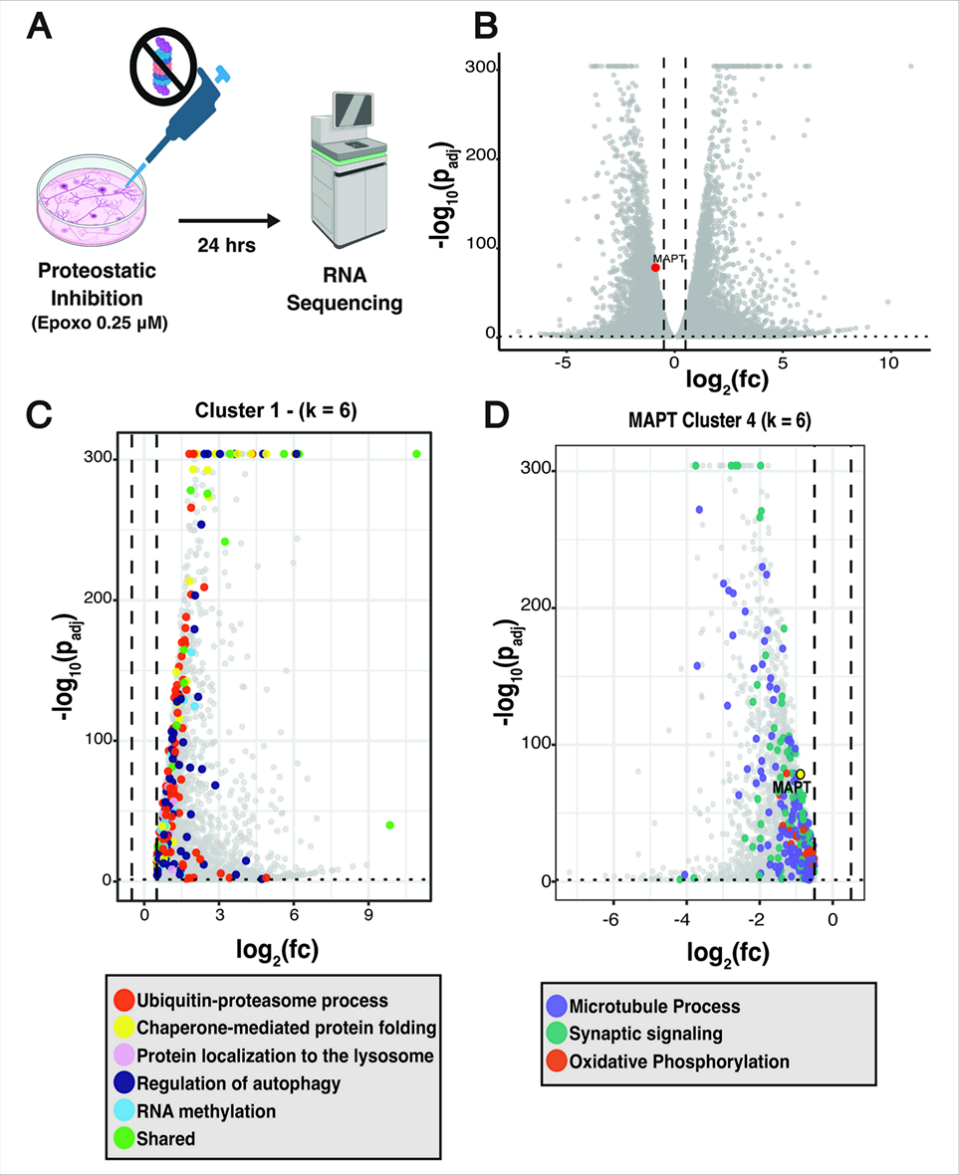
RNA Sequencing of UPS-Inhibited Neurons Triggers Activation of Proteostasis Quality-Control Mechanisms and a Suppression of MAPT-linked Neuronal Functions. **A**, Experimental design: iPSC-derived neurons were treated with epoxomicin (0.25 μM, 24 h) or vehicle (n = 3 biological replicates per condition), followed by RNA-sequencing. **B**, Volcano plot of differential expression (DESeq2).. C, Cluster 1 contains a high density of up-regulated genes. Points are colored by significant GO:BP categories enriched within this cluster (Fisher’s exact test with FDR correction), including ubiquitin-proteasome, autophagy/lysosome, chaperone-mediated protein folding, regulation of autophagy, and RNA methylation; grey points denote other DE genes within cluster 1. **D,** The *MAPT*-containing cluster 4 is down-regulated and enriched for neuronal/energetic functions, including microtubule processes, synaptic signaling/vesicle cycling, and oxidative phosphorylation (colored); grey points denote other DE genes in cluster 4. Full cluster memberships, additional clusters, and complete GO term lists are provided in **Sup. Tables 2 & 3.** For volcano plots **B-D,** the x-axis shows log_2_ fold-change (epoxomicin vs control) and the y-axis shows −log_10_(adjusted p). *MAPT* is labeled. Vertical dashed lines indicate log_2_FC cutoffs (±0.5); the horizontal dotted line marks the FDR = 0.05 threshold.

Following treatment with 0.25 μM epoxomicin for 24 hours, we performed RNA sequencing on the treated neurons compared to vehicle-treated control neurons (Fig. 8A). Differential expression (DE) analysis revealed global changes in the transcriptome with 8811 genes significantly upregulated and 8461 genes significantly downregulated (padj < 0.05) (Fig. 8B; total RNA seq results in **Sup. Table 2**). We then grouped the DE genes by their expression pattern across samples (hierarchical clustering on z-score expression (k=6), we identified two modules with opposing directions, cluster 1 — the module with the highest density of strongly up-regulated genes — and cluster 4, which contains *MAPT* and concentrates many of the most significantly down-regulated genes (volcano plot: **Fig. S7**, heatmap: **Fig. S8**). Cluster 1 was upregulated and significantly enriched for proteostasis-related programs, including ubiquitin-proteasome, autophagy/lysosome, chaperone-mediated protein folding, RNA methylation, and related quality-control pathways (Fig. 8C). The *MAPT*-containing cluster (cluster 4) was down-regulated and enriched for neuronal functions/energetic functions, including microtubule organization/transport, synaptic signaling/vesicle cycling, and oxidative phosphorylation (Fig. 8D; all cluster results in **Sup. Table 3**). In parallel, analysis of a curated unfolded protein response gene set showed strong upregulation of ATF3, DDIT3, & CTH (**Fig. S9**), confirming that proteasome inhibition also activates ER-stress pathways. These cluster-level changes, together with the induction of proteostasis/stress programs, motivated the subsequent transcription factor analyses (HOMER/JASPAR/ChEA3).

### Integrated transcription factor analysis supports ETV1, E2F1, and TCF3 as top candidates of MAPT regulation.

**Figure 9:**
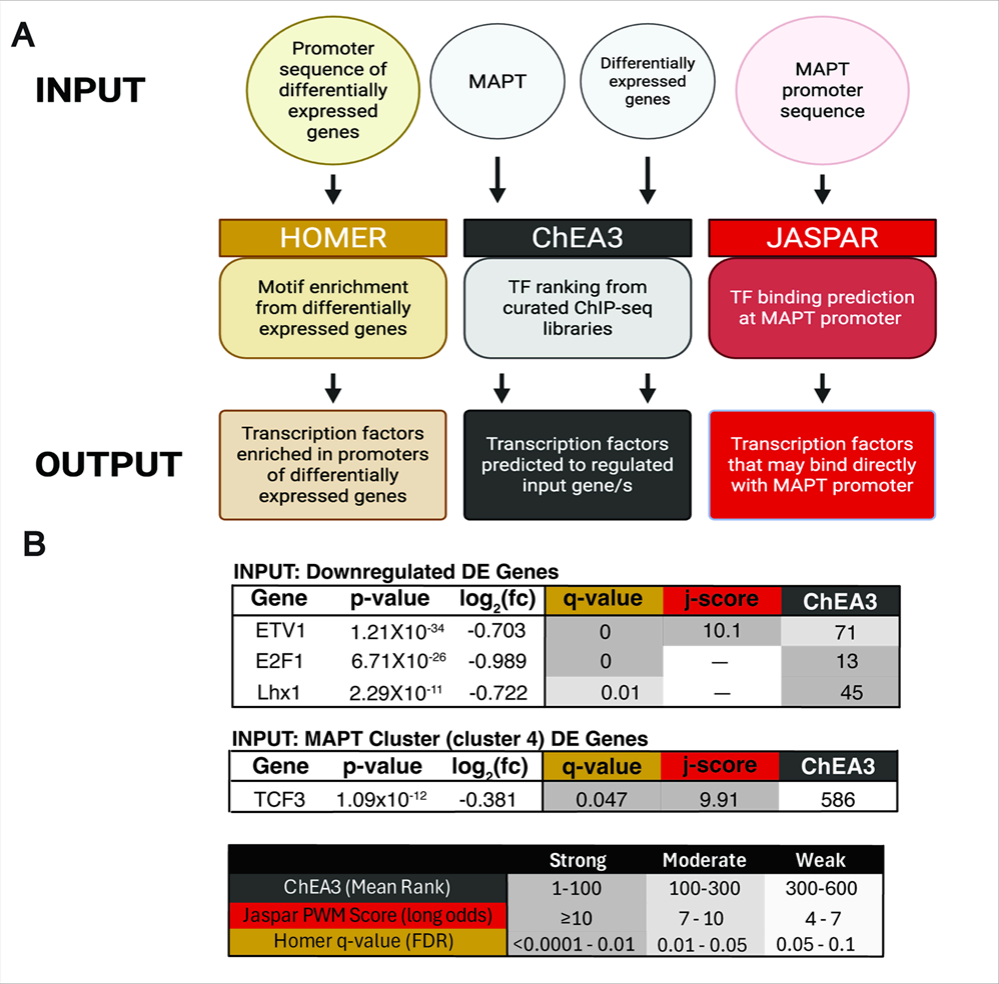
Integrated Transcription-Factor Prioritization for MAPT Regulation (HOMER + ChEA3 + JASPAR). **A,** Workflow schematic. Promoters of significantly down-regulated DE genes and of Cluster 4 genes (−300 to +50 bp relative to the hg38 TSS) were analyzed with HOMER for motif enrichment using GC/CpG-matched backgrounds; Cluster 4 was additionally tested against a custom background of down-regulated DE promoters excluding Cluster 4. The down-regulated and Cluster 4 DE gene sets were analyzed with ChEA3 (MeanRank across libraries). The *MAPT* promoter was scanned with JASPAR for candidate TF motif matches. **B**, Prioritized transcription factors with multi-tool support. The top tier highlights our results from analyzing significantly downregulated DE genes, indicating ETV1, E2F1, and LhX1 as top candidates. The middle tier highlights TCF3, the top candidate obtained from analyzing genes from the *MAPT* cluster (cluster 4). As indicated by the bottom tier, cells are shaded by evidence strength per tool; numbers report HOMER q-value, JASPAR PWM score, and ChEA3 Mean Rank. A transcription factor was retained if supported by ≥2 tools. Evidence was categorized as strong/moderate/weak using thresholds shown in key (lower HOMER q-values and ChEA3 ranks indicate stronger support; higher JASPAR j-score indicates stronger motif matches). See **Sup. Tables 4–6**. for full results list.

Using HOMER motif enrichment on promoters of differentially expressed downregulated genes identified significant enrichment for ETV1 (q-value = 0), E2F1 (q-value = 0), and Lhx1 (q-value = 0.01); ChEA3 ranked E2F1 and Lhx1 as among top regulators of the downregulated program (E2F1 rank = 13; Lhx1 rank = 45). Homer enrichment and ChEA3 ranking performed on differentially expressed genes specific to the MAPT cluster (cluster 4) identified TFC3 (E2A) as a candidate for *MAPT* regulation (q-value = 0.047; rank = 586). Direct motif scanning of the MAPT promoter (−300:+50 and −1000:+100, hg38) using motif PWMs from our HOMER results found no high-scoring matches for E2F1, ETV1 (ETS), TCF3/E2A, or LHX1 (all windows: 0 hits at HOMER’s default threshold) JASPAR scanning of the same sequence returned a high-scoring ETV1-like site (Jaspar PWM =10.1), and a moderate score for TCF3 (Jaspar PWM = 9.91) consistent with PWM/threshold differences across databases (Fig. 9). Together, these data support program-level regulation by the E2F (with TFDP), ETS (e.g., ETV1/ELF1/GABPA), and E-protein (TCF3/TCF4/TCF12) families, while MAPT itself likely lacks a strong proximal binding site and may be regulated distally or indirectly. All HOMER results in **Sup. Table 4**, all ChEA3 results in **Sup. Table 5**, and all Jaspar results in **Sup. Table 6**.

## Discussion

In this study, we demonstrated that in human iPSC-derived forebrain neurons, inhibition of the ubiquitin-proteasome system (UPS) or autophagy did not increase endogenous tau; instead, proteasome inhibition reduced total tau protein, accompanied by decreased *MAPT* transcription. When tau was expressed from a CMV promoter (decoupling it from the endogenous *MAPT* promoter), UPS or autophagy inhibition increased tau abundance, consistent with impaired clearance. RNA sequencing following proteasome inhibition showed broad induction of proteostasis/stress programs and a coordinated downregulation of neuronal modules including a microtubule/cytoskeleton cluster that contained *MAPT*. In human tissue, representative confocal images revealed local tau overlap with proteasome-positive regions in the AD cortex, with a more modest association with lysosomes; fetal and adult control fields showed limited apparent overlap. Integrated transcription factor analysis (HOMER/JASPAR/ChEA3) nominated ETV1, E2F1, Lhx1, and TCF3 as candidate regulators of *MAPT*-linked programs.

We observed upregulation of genes associated with the unfolded protein response (UPR) (**Fig S9**), which indicates activation of the UPR,and reduced tau in UPS-inhibited neurons (Fig 5A, C). However, when we expressed tau via a constitutive CMV promoter and inhibited the UPS, we observed tau accumulation due to impaired degradation (**Fig.6 C-D, Fig.7 A**). This is important because if *MAPT* downregulation were primarily a consequence of UPR-mediated translation attenuation, tau protein levels would be expected to decrease irrespective of promoter context. Instead, the promoter bypass experiment supports a locus-specific transcriptional brake on *MAPT* engaged during proteasome stress. Additionally, *MAPT* downregulation occurred outside of this canonical UPR signature and clustered instead with a neuron/microtubule cluster, highlighting that its regulation is not part of the canonical upregulation program that defines the UPR (Fig. 8D). While UPR signaling may contribute indirectly to MAPT repression, our integrated TF analysis (HOMER enrichment of DOWN promoters, ChEA3 regulator ranks, and JASPAR scans) points instead to the E2F–TFDP, ETS (e.g., ETV1/ELF1/GABPA), and E-protei**n** (TCF3/TCF4/TCF12) families as the primary regulators of the MAPT-downregulated program (Fig. 9).

Prior iPSC-neuron studies reported tau accumulation with proteasome inhibitors. Most recently, Hesieh et al. examined response to proteasome inhibition in iPCS-derived neurons from a cohort of patients with varying degrees of AD pathology and cognitive impairment, observing an increase in tau expression [15]. Their results are, however, not directly comparable to our own since they used lines with varying degrees of baseline UPS function, including several from patients with known Alzheimer’s disease, examined neurons at later stages of development, and focused exclusively on heavy molecular weight phosphorylated tau rather than total tau expression. While these findings differ from our own, fundamentally, our study sought to identify mechanisms preventing tau toxicity in developing neurons, while theirs sought to identify mechanisms for failure in AD. In agreement with our findings, recently reported work by Lines et al. showed no changes in total tau levels using wildtype neurons [24]. While our CMV-tau experiments were able to reproduce tau accumulation under UPS/autophagy inhibition, this required forced transcription of *MAPT* under the CMV promoter, implicating reduced clearance as the driver (Fig. 6 & Fig. 7). However, the fall in endogenous tau in unmodified neurons that we observed likely reflects an adaptive transcriptional response that is present in these developing neurons that may be weakened or absent in disease contexts or later maturation (Fig. 5C).

Together, the data support a model in which proteasome impairment triggers the activation of proteostasis and stress pathways, as well as transcriptional downregulation of the neuron/microtubules module, which includes *MAPT*. In parallel, tau physically engages proteostasis networks (co-IP/MS – Betters et al. 2023) (Fig. 1), and in AD tissue, it is frequently nested within proteasome-positive regions (Fig. 2). Integrating motif enrichment (HOMER), regulator ranking (ChEA3), and promoter scans indicate that this program is best explained by coordinated action of E2F–TFDP, ETS, and E-protein transcription factors (Fig. 9). Notably, the MAPT core promoter lacks a strong proximal site for these factors by HOMER, implying regulation through distal elements or indirect mechanisms. Under this model, developing neurons impose a stress-contingent transcriptional brake on MAPT; when tau is forced from a constitutive promoter, that brake is bypassed and tau accumulates under UPS or autophagy inhibition (Fig. 6 & Fig. 7).

Our study provides valuable insight into how proteasome and autophagy inhibition impact neuronal gene expression using human IPSC-derived neurons. However, it is limited by our use of a single female stem cell line, which allowed for controlled and consistent comparison across conditions but may limit the generalizability of our findings across different genetic backgrounds or sexes. Additionally, in this study, we utilized pharmacological inhibitors to modulate degradation pathways, which are effective and widely used tools. While epoxomicin, our proteasome inhibitor, is considered a very clean pharmacological inhibitor, pharmacological inhibitors may have off-target effects that must be considered [26]. We also used a CMV promoter, which is strong, but not expression-invariant; although tau increased with UPS/autophagy inhibition in the CMV-tau condition, supporting a clearance effect, future work should verify transgene mRNA stability across treatments and/or replicate with a non-CMV promoter. It is essential to note that our differentiation method, although robust, was not an NGN2-driven protocol, which yields a more transcriptionally and functionally mature, homozygous population. Our differentiation protocol resulted in a more heterogeneous neuronal population, which may introduce variability in the cellular responses. Yet, we have shown that our population can differentiate into mature neurons with electrophysiological activity (Fig. 3). Although the cultures were predominantly neuronal, a low level of astrocytic contamination, which is unavoidable with these protocols, was present and could influence gene expression profiles. Despite these considerations, the study offers a controlled platform for mechanistic exploration of proteostasis disruption in human neurons and sets the stage for future validation across additional models. In future studies, it will be important to mechanistically perturb E2F1, EVT1, Lhx1, and TCF3 to test their effects on *MAPT* and to examine whether adaptive *MAPT* downregulation is present in aged neurons and in AD-derived lines.

In conclusion, we propose that developing human neurons deploy an adaptive, transcription-level response to proteostatic stress inhibiting the expression of mRNAs for potentially toxic proteins such as tau. This response limits tau burden when degradation pathways are compromised, offering a mechanistic counterpoint to the accumulation observed in disease settings. Understanding how this transcriptional safeguard is engaged, and why it might fail, may suggest strategies to preserve tau proteostasis early in neurodegeneration.

## Supplementary Material

Supplementary Files

This is a list of supplementary files associated with this preprint. Click to download.

• 9.9.25SupplementalInformationShin.docx

• Sup.Table6JASPAR7.28.25matchedtfsMAPTpromoter.xlsx

• Sup.Table3AllClustersGOBP.xlsx

• Sup.table1MassSpec.xlsx

• Sup.Table4HOMERknownresults.xlsx

• Sup.Table2RNASeqDataandGOBP.xlsx

• Sup.Table5AllChEA3Results.xlsx

• FigS5UncroppedWesternBlots.docx

• Tables.docx

## Data Availability

All relevant data is included in the manuscript and supplemental Information.

## Abbreviations

AD: Alzheimer’s disease
UPS: ubiquitin-proteasome system
AU: arbitrary units
DIF: differentiation
ICC: immunocytochemistry
BCA: Bicinchoninic Acid
DMEM: Dulbecco’s Modified Eagle Medium
iPCSs: induced pluripotent stem cells
NPCs: neuronal progenitor cells
PBS: phosephate buffer saline
